# Heat Illness and Extreme Weather Health Literacy: Communication Preferences and Effectiveness for Patients Living in Climate-Change-Vulnerable Communities

**DOI:** 10.3390/ijerph22030434

**Published:** 2025-03-14

**Authors:** Todd L. Sack, Aran R. Thiravialingam, Carlos Suanes Zubizarreta, Robby Felix, Rita Kanazeh, Innah Lachica, Eddy Hernandez Cuesta, Alan Martin, Frederick Anderson, Cheryl Holder

**Affiliations:** 1 Department of Translational Medicine, Herbert Wertheim College of Medicine, Florida International University, Miami, FL 33199, USA; 2 Herbert Wertheim College of Medicine, Florida International University, Miami, FL 33199, USA; athir006006@med.fiu.edu (A.R.T.); csuan001@med.fiu.edu (C.S.Z.); ehern236@med.fiu.edu (E.H.C.); amart668@med.fiu.edu (A.M.); clholder@fiu.edu (C.H.); 3 Graduate School of Biomedical Sciences and Professional Studies, Drexel University, Philadelphia, PA 19104, USA; robbygallandfelix@gmail.com; 4 Robert Stempel College of Public Health and Social Work, Florida International University, Miami, FL 33199, USA; rkana003@fiu.edu; 5 School of Medicine, St. George’s University, Saint George’s P.O. Box 7, Saint George’s Parish, Grenada; ilachica@sgu.edu; 6 Department of Medical Education, Herbert Wertheim College of Medicine, Florida International University, Miami, FL 33199, USA; fwanders@fiu.edu

**Keywords:** health education, health promotion, health literacy, climate change, extreme heat, heat-related illness, environmental justice, health emergency, behavioral change

## Abstract

Health professionals are trusted information sources and could be valuable for improving climate change health literacy. Few studies address teaching patients about health risks associated with climate change, and no studies have focused on the medical office waiting room as a teaching site for populations from heat-vulnerable neighborhoods. We gave adult patients in primary care office waiting rooms printed teaching materials about heat-related illnesses. We asked them to read these at home and then complete an online confidential survey concerning their preferences among teaching methods and their preferences for communication during health emergencies. Ninety-one surveys were received from patients residing in heat-vulnerable neighborhoods. Patients liked receiving information in waiting rooms. Printed brochures were favored statistically by patients, but other teaching methods that are feasible for waiting rooms also rated well, including single-page printed fliers, posters, and video screens. Digital options were far less favored. We conclude that printed teaching materials may improve decisions that impact human health. The medical office waiting room appears to be an accepted, time-efficient, and effective site to communicate knowledge on climate change and health. Additionally, medical offices could play a role supporting government agencies to communicate with patients during weather-related health emergencies.

## 1. Introduction

According to the 2023 report of the Lancet Countdown, mortality caused by extreme heat events increased by 53.7% from 2000 to 2018 among people over 65 years old, resulting in 296,000 deaths worldwide in 2018 [1]. Extreme storms and their long-term impacts on the health of communities are substantial, and these health effects may even exceed those of heat [2]. These risks are particularly relevant in tropical south Florida, our study location, where residents face multiple climate-related health threats.

Climate change represents one of the most significant public health challenges of the 21st century, posing widespread threats to human health globally [1]. Beyond the impacts of extreme heat and storms are profound health impacts resulting from water shortages, increased incidences of vector-borne diseases, malnutrition, psychological stresses, population displacement, loss of life, and wars [1]. These health burdens disproportionately affect marginalized communities and older adults, making climate change mitigation central to health equity and social justice [3].

“Climate change health literacy” means being knowledgeable about the causes of climate change, the consequences for human health, the personal precautions needed to avoid climate-related illness, and the steps needed at a societal level to mitigate climate change [4]. Families may experience multiple health benefits from increased climate change literacy. For example, they will know how to protect themselves from heat-related illness, be prepared for extreme weather events, decrease their exposure to indoor air toxins through better choices of energy and chemicals, make more visits to public parks, and choose healthier, plant-based diets [5,6].

Many sizable health professional societies, including the British Medical Association, the American Medical Association, and the American Academy of Family Physicians, have declared climate change to be a significant health risk, yet only a few provide any information to practitioners for their offices or for patients to take home [7,8]. Some resources are accessible only to dues-paying physician members [8]. Additionally, public health agencies and media outlets have not effectively delivered clear, unbiased information to guide climate-related decision making at household and community levels [9].

Surveys report that health professionals are among the most trusted members of society. Nurses, doctors, and other healthcare providers are generally ranked among the top five professions, trusted more even than clergy, journalists, or elected leaders [10]. A 2024 non-peer-reviewed survey of 1006 adult Americans found that 69% of those surveyed trust their health professionals “some or a lot” as sources of information on climate change [11]. Other surveys tell us that a large majority of nurses and physicians consider climate change to be a significant threat to human health and that health professionals have a responsibility to explain the health risks of climate change and even to advocate in the public arena [12]. Increasingly, professionals are recognizing the impacts of climate change to be an important social determinant of health outcomes [12]. Even with this high level of trust and professional awareness of the problem, there remains a significant gap between potential and actual climate change teaching in healthcare settings.

There are few documented examples of clinicians communicating with patients about any environmental risk, including climate change [13]. In two studies where pediatricians spoke with their patients about climate change, patients reported being pleased to receive the information [13,14]. The first surveyed 371 largely middle-class parents in one U.S. pediatric office who were asked about climate change communication by their pediatrician [13]. Eighty percent of parents agreed or strongly agreed that the impact of climate change on their child’s health should be presented, and yet only 4% recalled receiving this information in the prior twelve months. For parents with children old enough to understand, 89% wanted their doctor to explain what to do if a child is feeling stressed about global warming. The majority of the parents wanted their health professionals to explain how to protect against the health risks of climate change (57%) and how families can mitigate against climate change (54%). Despite this, parents are less interested in becoming climate advocates; only 38% would like their doctor to explain how to talk to decision makers.

The second study, also from a single pediatric practice caring for middle-class children, suggests that even a brief clinical intervention can be effective climate change communication [14]. Lewandowski et al. reported the results of one physician in a Wisconsin practice delivering a standard forty-five-second message on climate change and health as part of the pediatric appointment [14]. Following the visit, 138 parents or patients answered an anonymous survey about their experience. A large majority of the respondents appreciated the information and reported that they would make changes at home as a result of the office visit. This was true for self-described political liberals, moderates, and conservatives. One parent said about the encounter, “[I] appreciate [my doctor] creating awareness, and trust [my doctor’s] guidance on all topics”. This finding aligns with broader research on healthcare communication effectiveness. Boland et al. concluded similarly: “As highly trusted and underutilized resources, physicians are uniquely placed to educate patients and encourage public health efforts in this area” [15]. Nonetheless, it is doubtful that many clinicians are willing to follow the example of Lewandowski et al. to use scarce examination room time to teach about climate change.

The healthcare office waiting room is a well-established and accepted site for health promotion. The average U.S. resident will visit an office-based physician two to three times per year, according to the Centers for Disease Control and Prevention, where the average wait time will be 18 min per visit [16]. Patients are receptive to health information in the form of brochures, posters, and video screen presentations [17]. After being provided in the waiting room with an information toolkit on home safety, 91% of patients surveyed said that the intervention resulted in a better waiting room experience [18].

A study conducted in family practice office waiting rooms in Belgium among largely Caucasian and “socioeconomically advantaged” patients found that 94% of patients stated that they read the health information “leaflets” that were displayed in the waiting room [19]. Furthermore, 45% of survey respondents took leaflets home, 42% shared the content of brochures with others, 34% stated that brochures helped improve their health knowledge and self-management, and 19% discussed them with their doctor.

The medical office waiting room has not been studied as a site for teaching about climate change and health. Moreover, communities highly vulnerable to climate change have not been well-studied. The present study aimed to examine how patients in climate-vulnerable communities prefer to receive climate change health information from healthcare providers. Specifically, we investigated the following.

1. Patient receptiveness to climate change health information in the waiting room;
2. Effectiveness of printed educational materials provided in waiting rooms;
3. Patient preferences among ten teaching methods that are available to healthcare offices;
4. Preferred communication channels during an extreme heat or other weather emergency.

## 2. Materials and Methods

### 2.1. Study Design and Ethics

The study was conducted in Miami, Florida, USA in 2023 and 2024. It used an anonymous online survey methodology to assess the opinions of patients in waiting rooms to assess how they wish to receive information from their clinicians about heat-related illnesses and during an extreme weather health emergency. The focus was to survey patients or family members visiting primary care practices located in zip codes throughout Miami–Dade County with increased risk of heat-related illness, hospitalizations, and emergency department visits.

The protocol encompassed seven sequential steps: 1. design and selection of the educational items to be studied, 2. selection of medical office waiting rooms as study sites, 3. distribution of educational items and survey instructions to patients in waiting rooms, 4. study of the educational items by patients once they return home, 5. completion at home of the online survey, 6. removal of any potentially identifying information from the survey results, and 7. data analysis by the co-authors.

To locate clinics for this study, climate-vulnerable communities were identified by zip code using the 2022 Florida Heat Vulnerability Index [20]. The Index was developed by the Florida Department of Health based on 2021 census data and using socioeconomic criteria that predict a neighborhood’s medical vulnerability to heat-related illnesses for each zip code. The Index rates neighborhood heat vulnerability using a scale that ranges from level 1 for the least vulnerable to level 5 for those most vulnerable.

Medical clinics in Index levels 3, 4, or 5 in a single contiguous area of the Miami–Dade County area were prioritized for this study. In order to try to reach individuals who are the most vulnerable to climate change in these neighborhoods, the protocol primarily studied patients in waiting rooms in Federally Qualified Health Centers (FQHCs), which in most regions of the United States are the principal “safety net” health providers serving people who often are impoverished, unhoused, uninsured, or underinsured [21]. Ninety primary care health facilities were contacted by phone or email to inquire about their interest in participating in the study. Seven organizations agreed to participate, including four FQHCs, two hospitals that own primary care clinics, and one independent, free clinic. A total of twenty-seven clinical sites were utilized, located in seventeen unique zip codes within Heat Index levels 3, 4, or 5.

Each of the seven potential participating organizations was visited by one co-author (C.H.), who met with the manager to obtain permission to include the organization’s practices in the study. The clinicians and other staff at each study site were offered a brief presentation about the study, but these individuals played no direct role in the protocol.

Thirteen co-authors, pre-medical undergraduate students, medical students, or advanced nurse practitioners served as trained volunteers to visit the study site waiting rooms to distribute educational items to patients or family members in waiting rooms. Prior to distributing educational information, prospective volunteers were required to complete a 3-part training session and submit a video recording to demonstrate their knowledge and preparedness to distribute the educational items according to the protocol.

Adult patients or family members in waiting rooms were approached by a volunteer and recruited through face-to-face invitation using convenience sampling. Each patient received a brief talk (under one minute) on the importance and prevention of heat-related illness. Potential survey respondents were offered a gift bag containing three printed educational items on heat-related illnesses: a trifold brochure titled “Surviving Extreme Heat” that was developed by My Green Doctor [22], a one-page flier titled “Heat Stress Overview” that was created by the US National Institute for Occupational Safety and Health [23], and a one-page flier titled “Staying Safe in Hot Weather” that was created by the US National Institutes on Aging [24]. These items are accessible online and in the Supplementary Materials. The gift bag included the three printed educational materials, cooling tools (a fan and a towel), a refrigerator magnet, and a survey instruction card. Potential survey participants were asked to complete the survey at home and told of the option of receiving a USD 10 gift card. Completion of the survey was not required for receipt of the gift card. All printed materials and the survey were offered in English and Spanish.

The printed instruction card directed study candidates to read the three educational materials at home and then to use the provided URL link or QR code to begin a fourteen-question survey that could be accessed by cell phone or larger format computers.

The survey questions and survey results are provided in the Supplementary Material section. Qualtrics was used to create and administer the survey, for data storage, and data analysis (https://www.qualtrics.com, accessed on 7 March 2025). The survey employed plain language and required under five minutes to complete (authors’ observations). Study patients were instructed to enter a 4-digit number unique to each instruction card to ensure one-time survey access and prevent duplicate responses. Any potentially identifying patient information was deleted from the database by the lead author (T.L.S.) prior to data analysis. Informed consent was obtained in the first survey question.

The protocol was reviewed by the Institutional Review Board of Florida International University, Miami, Florida, and it received approval for exempt human subject research on 20 September 2023 as study IRB-23-0463.

### 2.2. Subject Eligibility

Candidates needed to be English- or Spanish-speaking adults 18 years old or older in waiting rooms of primary care medical offices in climate-vulnerable neighborhoods of Miami–Dade County, Florida, USA.

### 2.3. Statistical Analysis

Statistical analysis was performed by a professional statistician and using the Two Sample Z Test to compare the independent proportions of each teaching method selected, with *p*-values reported to indicate significant differences. Calculations were confirmed using the Sapio Research online tool (https://sapioresearch.com/significant-difference-calculator/, accessed 7 March 2025).

## 3. Results

### 3.1. Participation and Subject Characteristics

Over an eighteen-month period during 2023 and 2024, thirteen co-authors and trained volunteers delivered the three heat illness educational items inside of gift bags to 439 adults in the waiting rooms or waiting areas of twenty-seven primary care practices in seventeen different Miami–Dade County zip codes and at Heat Index risk levels 3, 4, or 5. Two of the practices were mobile clinics, customized vehicles with standard medical equipment allowing the clinicians and staff to provide services to multiple addresses and reach underserved communities.

**Figure 1:**
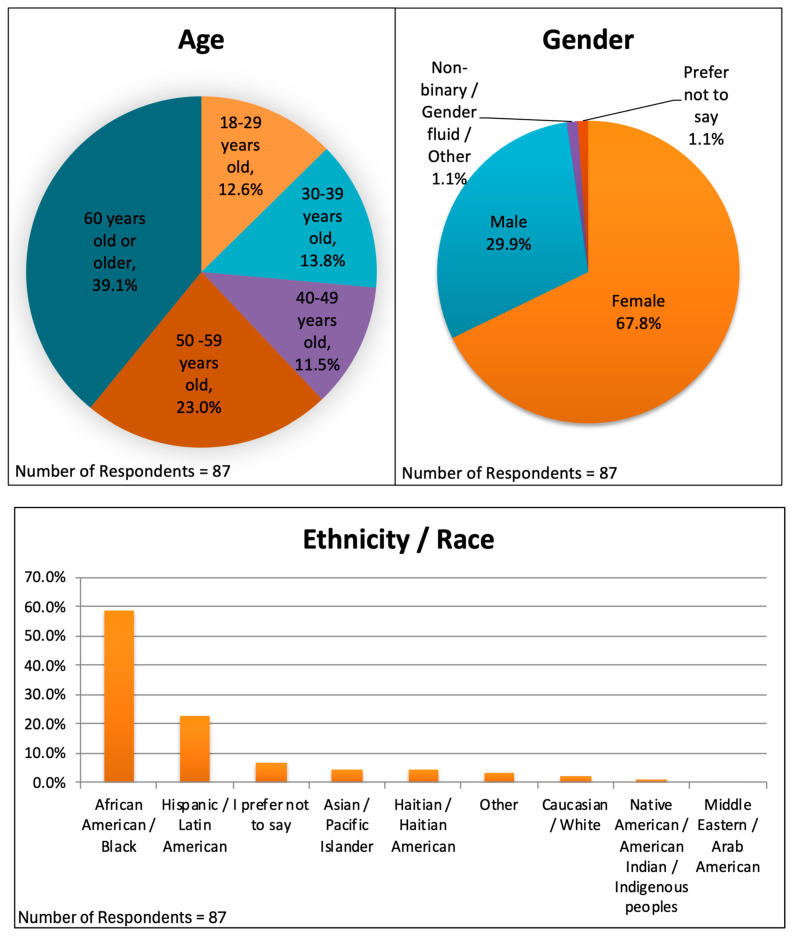
Demographics, including age, gender, and ethnicity/race distributions, for 87 survey respondents.

Ninety-four surveys were completed, but three of these were test surveys by the co-authors, leaving ninety-one surveys for data analysis, a 21% response rate. The complete data are provided in the Supplementary Material. Not every question received ninety-one responses, because some patients chose not to respond to certain questions and because seven respondents were not allowed to answer some questions because they stated that they had not read the educational items. The demographics of the respondents are shown in Figure 1. They included 59 females (67.8%), 26 males (29.9%), 1 gender non-binary person (1.1%), and 1 person who preferred not to say (1.1%). Eleven respondents were 18–29 years old (12.6%), twelve were 30–39 (13.8%), ten were 40–49 (11.5%), twenty were 50–59 (23.0%), and thirty-four were 60 years old or older (39.1%). Fifty-one of the respondents (58.6%) identified as African American, twenty (23.0%) as Hispanic or Latin American, four (4.6%) as Haitian or Haitian American, four (4.6%) as Asian or Pacific Islander, three (3.4%) as Other, two (2.3%) as Caucasian or White, and one (1.1%) as Native American, American Indian, or Indigenous Peoples. Six (6.9%) chose not to disclose their racial or ethnic background. Eleven percent of surveys were completed in Spanish.

**Figure 2:**
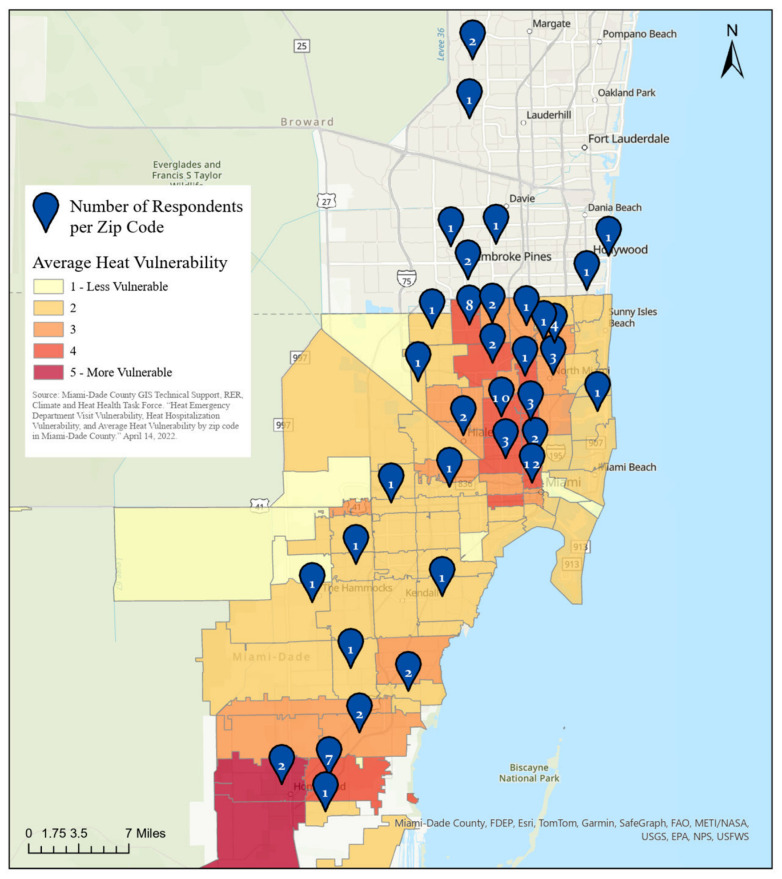
Number of survey respondents living within each zip code (blue balloons) overlaying a map displaying the Florida Heat Vulnerability Index for Miami–Dade County. Number of respondents: 86.

Our study was conducted in waiting rooms of primary care medical practices located in heat-vulnerable neighborhoods as defined by the Florida Heat Vulnerability Index. Figure 2 confirms that most of the patients responding to the survey also resided within recognized heat-vulnerable neighborhoods, those neighborhoods rated 3, 4, or 5, where 5 is the most vulnerable classification [20]. Five of ninety-one respondents did not provide their residential zip code.

### 3.2. Interest in Receiving Climate Change Information

**Figure 3:**
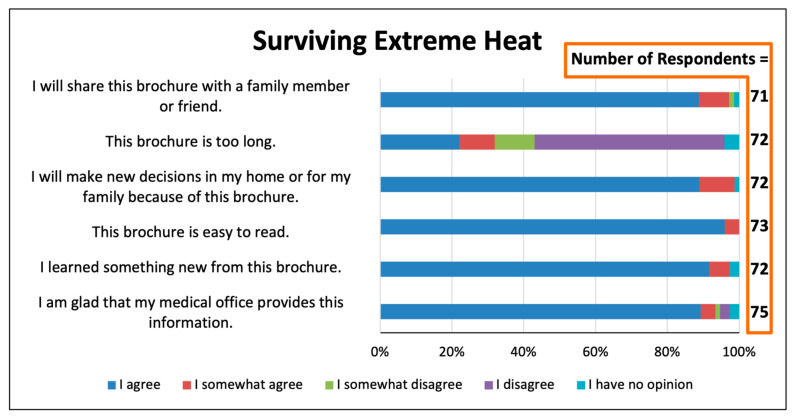
Responses concerning the trifold brochure, “Surviving Extreme Heat”.

**Figure 4:**
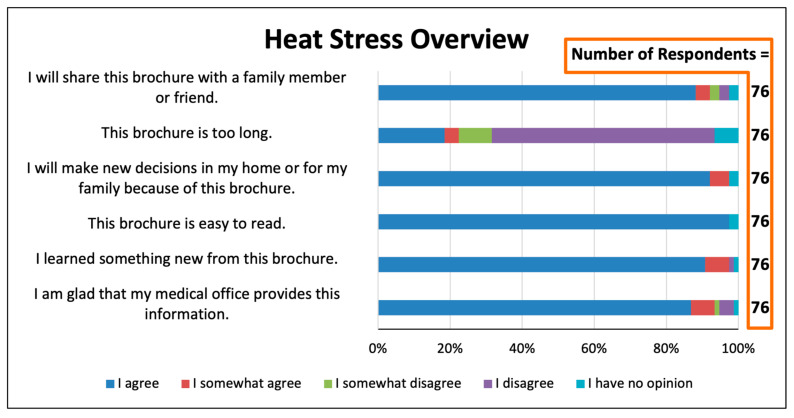
Survey responses concerning the one-page flier, “Heat Stress Overview”.

**Figure 5:**
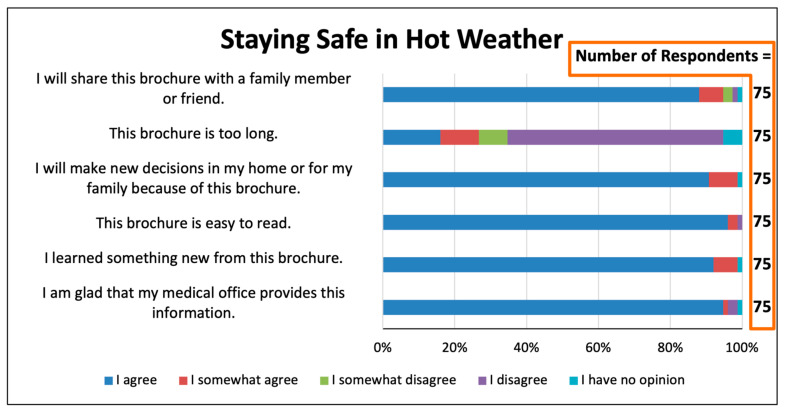
Survey responses concerning the one-page flier, “Staying Safe in Hot Weather”.

**Figure 6:**
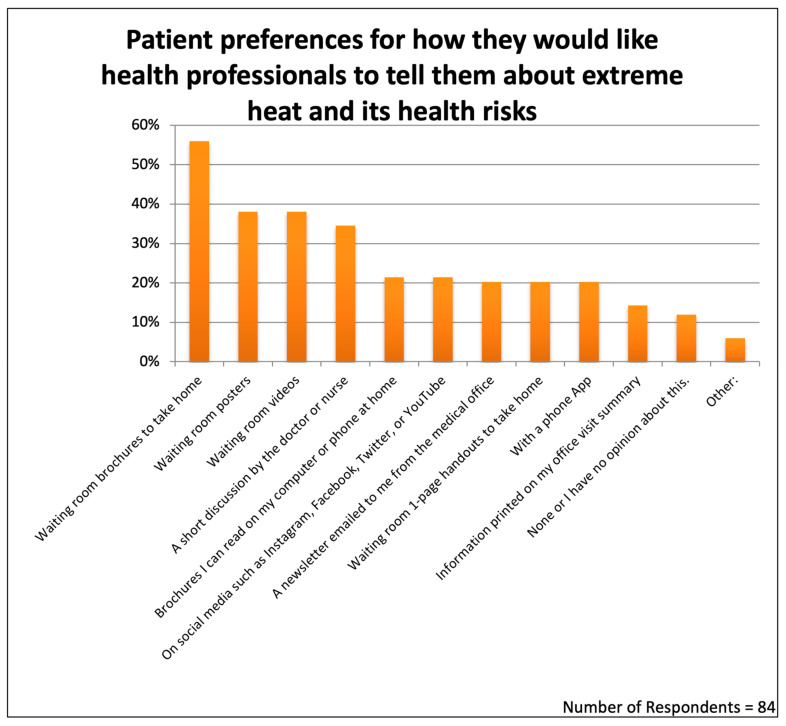
Patient preferences for receiving information about extreme heat and health.

**Figure 7:**
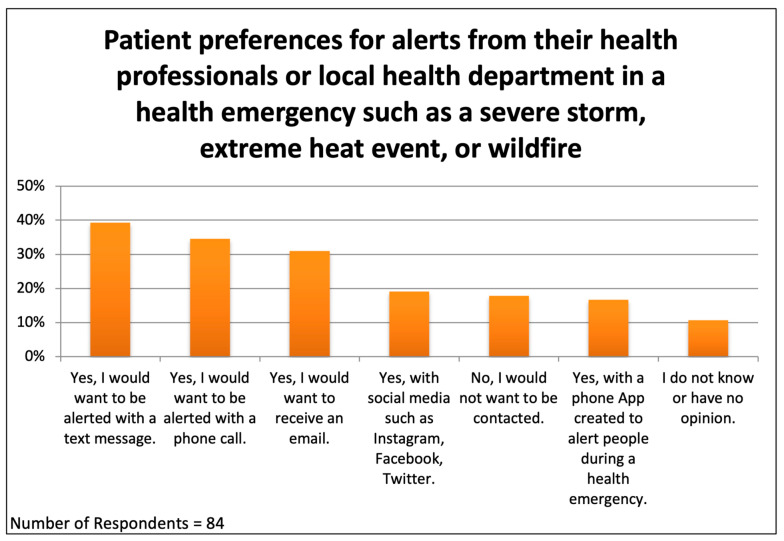
Patient preferences for receiving health alerts from their health professionals or local health department during a climate change health emergency.

Patients responded very favorably to questions about whether medical professionals should teach patients about climate change and health (Figure 3, Figure 4 and Figure 5). For the three educational items, 85–87% agreed with the statement “I am glad that my medical office provides this information”. For each of the items, only a total of 2–3% disagreed with this statement or had no opinion. When asked how they wished to be told about extreme heat and its health risks, 34.5% stated they “would like to have a short discussion by the doctor or nurse”, and just 11.9% stated that they would not want to learn about this from the medical office or had no opinion (Figure 6). More than one-third would like to be alerted by telephone or text message during a climate change health emergency (Figure 7).

### 3.3. Evaluation of the Six-Page Trifold Brochure on Heat-Related Illnesses

The same six questions were asked for each of the three educational items. Respondents were only allowed to answer questions for educational items that they stated they had read.

The six-panel trifold brochure is a teaching tool used widely in healthcare and other fields but that has not been studied previously for topics of climate change in health. The brochure “Surviving Extreme Heat” [22] was well-received (Figure 3). Ninety-six percent found the brochure to be “easy to read”. Although it is longer than the one-page fliers, only 22% agreed and 9.7% agreed somewhat with the statement “the handout is too long”.

The brochure appears to be an effective teaching method for this topic and audience, with 89% stating they would share the brochure with a family member or friend, 89% reporting that the brochure would lead them to make new decisions in the home or for their families, and nearly every respondent agreeing that they learned something new.

### 3.4. Evaluation of Educational Fliers on Heat-Related Illnesses

Seventy-six respondents indicated that they read the one-page flier, “Heat Stress Overview” (Figure 4). The vast majority agreed that they found this item easy to read, that the handout will prompt them to make new decisions in their home or in their family, that they learned something new, and that they will share the handout with family and friends. Only 18% agreed with the statement that “that the handout is too long”, a smaller figure than was the case for the six-page brochure. Notably, more that 86% agreed with the statement “I am glad that my medical office provides this information”.

Similarly positive responses were received from 75 participants for the one-page flier, “Staying Safe in Hot Weather” (Figure 5). Respondents reported overwhelmingly that this flier was easy to read, that they would share the flier with others, that they would make new decisions in their home or for their family because of the flier, that they learned something new, and that they were glad that the medical office provided the flier.

### 3.5. Patient Preferences Among Ten Teaching Methods Available to Medical Offices

The survey included one question asking patients how they would like their medical professionals to tell them about extreme heat and its health risks. This question offered ten possible teaching methods and allowed respondents to choose as many as they wished (Figure 6).

The teaching modality selected by the largest number of respondents was printed brochures, chosen by 56% of the patients. This choice of printed brochures was statistically compared with the other teaching options. The *p* values for choosing “Waiting room brochures to take home” versus “Waiting room posters” was 0.020; for choosing brochures over “Waiting room videos”, it was 0.020; for choosing brochures over a “Short discussion by the doctor or nurse”, it was 0.005; for choosing brochures over “Brochures I can read on my computer or phone at home”, it was 0.000004; for choosing brochures over ”On social media”, it was 0.000004; for choosing brochures over “Newsletter emailed from the medical office”, it was 0.000002; for choosing brochures over “Waiting room 1-page handouts to take home”, it was 0.000002; for choosing brochures over “With a phone App”, it was 0.0000012; and for choosing brochures over ”Information printed on office visit summary”, it was 0.000000015. The printed brochure included a QR code for reading the trifold brochure online at home using a computer or smartphone. We do not know how many patients took advantage of the QR code, but online access to the brochures was a preference selected by only 21% of the respondents.

An equal proportion of respondents, 38.1%, favored seeing waiting room posters or waiting room videos. Less attractive seemed to be receiving information through social media (21.4%), a newsletter mailed to the home (20.2%), information delivered by a phone app (20.2%), or information included in the printed summary following the office visit (14.3%). Only 11.9% answered with “None or I have no opinion”.

### 3.6. Patient Preferences for Notification During a Climate Change Health Emergency

The survey asked patients how they would like to be contacted during a “health emergency such as a severe storm, extreme heat event, or wildfire”. The respondents could choose as many options as they wished; eighty-four responded to this question (Figure 7). Surprisingly, 17.9% told us that they would not want to be contacted or had no opinion. The most popular method of communication was text messaging, chosen by 39.3% of respondents, followed by “a phone call” (34.5%), an email message (31%), using social media (19%), and using “an app created to alert people during a health emergency” (16.7%). Respondents were not asked to rank their choices, but it appears that no single modality would be effective for reaching everyone at risk. It is difficult to exclude a Primary Effect Cognitive Bias, a tendency for people to choose the first few items in a list, but the order of presentation of the options in the survey questions was not the order depicted in Figure 6 and Figure 7 (see Supplementary Materials).

### 3.7. New Decisions Made at Home as a Result of the Educational Items

**Figure 8:**
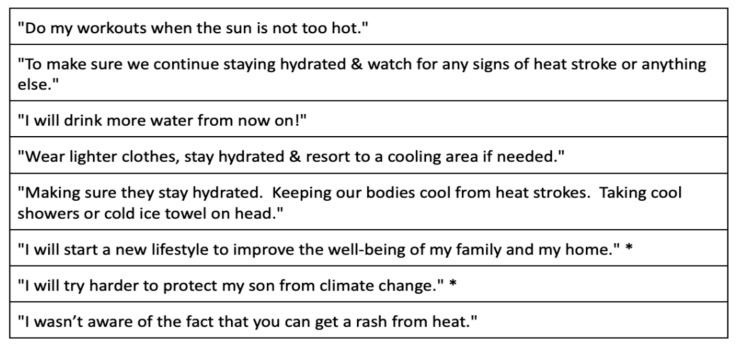
Selected participant responses to “To tell us of any new decisions you may make in your home or for your family because of the brochure or handout you were given” (* indicates translation from Spanish).

A single survey question provided a free text space for respondents “to tell us of any new decisions you may make in your home or for your family because of the brochure or handouts you were given”. Of the sixty-two responses to this question, many simply expressed appreciation for the educational materials. Twenty-four described specific decisions that they planned; some of these are listed in Figure 8.

## 4. Discussion

Climate change poses significant health risks, creating an urgent need for effective education strategies to help families mitigate these risks and protect themselves [1,2,3]. Beyond the personal protection of their health, teaching families about solutions such as energy efficiency, safe uses of chemicals, renewable energy, reducing plastic waste, and adopting plant-based diets offers financial benefits to families. We speculate as well that improved climate literacy will strengthen community support for climate mitigation and adaptation policies across local, state, and national levels.

The current study is important to the topic of climate change and health education. Our findings extend in several ways the important results of Ragavan et al. [13] and Lewandowski et al. [14]. First, this is the first IRB-approved study of climate change education conducted in medical office waiting rooms. Second, climate change teaching in family practice offices has not been studied previously. Third, this is the first report from a highly climate-vulnerable community. Fourth, perhaps most importantly, we provide new insights from patients themselves on whether they wish to receive this information and how they wish to receive it. Fifth, our data indicate new cost-effective and time-effective educational opportunities for health professionals to help protect their climate change vulnerable patients.

### 4.1. Population Studied

We used the Florida Heat Vulnerability Index to identify high-risk neighborhoods [20]. To reach people who are likely the most vulnerable residents of those neighborhoods, we principally conducted the study at Federally Qualified Health Centers (FQHCs), free clinics, and mobile clinics, which in the United States are important “safety net” health providers serving patients who often are impoverished, unhoused, or uninsured [21]. One limitation of our study was that we did not sample clinics randomly from all of the clinics in the area; this meant that we cannot gauge the size of the population’s statistical universe. However, Figure 1 and Figure 2 confirm that our survey respondents reside in heat-vulnerable neighborhoods and that our data reflect diversity by age and gender. The respondents self-described largely as African American or Hispanic, which we know to be the preponderant ethnicities cared for at the locations we surveyed.

Patients self-describing as African American, Hispanic, Haitian, or Asian comprised more than 90% of our respondent sample. This is an important aspect of our study in that these communities are among those disproportionally affected by climate change [1,3] and because previous studies have not focused on the communication preferences of these groups.

The survey participation rate of 21% was lower than that found by Ragavan et al. (71%) and Lewandowski et al. (58.5%) [13,14]. The latter two studies were also climate change surveys of patients conducted in primary care offices, but our study faced the additional challenges of asking patients to read three educational items after returning home and then complete our online survey without the prompting of office staff. Although we could not study this directly, lower than average literacy rates may have added to the lower survey response rate. Another factor contributing to our low response rate may be related to our patients coming from historically disadvantaged communities. The historical distrust by those communities of health professionals and medical researchers has been well-documented [25].

### 4.2. Patient Receptiveness to Climate Change Health Information in the Waiting Room

We do not know of a previous study that has asked patients whether they favored receiving educational materials in the waiting room on the topic of heat-related illnesses, and none have focused on the opinions of residents of areas highly vulnerable to extreme heat events. For the educational brochure and for the two one-page fliers that we provided, 95% of respondents or more “agreed” or “agreed somewhat” with the statement “I am glad that my medical office provides this information” (Figure 3, Figure 4 and Figure 5). These results, which are consistent with those of Lewandowski et al., indicate that patients are likely to welcome receiving in waiting rooms information on topics of climate change and health that is evidence-based, non-political, and health-related [14].

### 4.3. Effectiveness of Educational Materials Provided in Waiting Rooms

Printed educational items, such as brochures and fliers, to encourage energy efficiency have been distributed in non-healthcare settings. Meta-analyses show these interventions to be somewhat effective at changing climate change mitigation behaviors [26]; topics of climate change and health have not been studied. In the present study, most respondents found our brochures and fliers to be “easy to read”, and most “will make new decisions in my home or for my family because of this brochure” (Figure 3, Figure 4 and Figure 5). The respondents’ specific “new decisions” reported in the final survey question are encouraging in this regard Figure 8). The obvious limitations of our study are that the survey size is small, the behavioral changes are self-reported, and they are not monitored over time. Nonetheless, these results of behavioral improvements are stronger than expected in the medical literature [26]. We believe that having the educational items provided by trusted health professionals [10] enhances the efficacy of educational tools to teach about heat illness, extreme weather, and other environmental health topics.

### 4.4. Patient Preferences for Printed Materials Among Ten Teaching Methods That Are Available to Healthcare Offices

In order to offer guidance to medical practices on how to teach their patients, we asked patients which of the ten teaching methods they favored. We were somewhat surprised to find that printed trifold brochures were the most frequently selected teaching method for the medical office, chosen by 56% of patients compared with just 21% choosing to read the same brochures on a phone or computer screen. This might be explained by the age of our patients, as 73.6% were 40 years of age or older. Clearly, it seems useful to offer patients printed brochures to take home and potentially be read by many family members. Posters, digital video screens in waiting rooms, and “a short discussion by the doctor or nurse” were also favored.

Our results have an added importance because they represent the opinions of three highly vulnerable groups: people 60 years of age and older, African Americans, and Hispanic Americans [1,3]. Our small sample size does not allow us to distinguish statistically between the preferences of these groups or among subgroups. For example, we cannot define whether younger African American patients compared with older ones prefer electronically delivered information, such as with QR codes, online newsletters, or social media. Future research could provide more precise comparative insights into the preferences of other ethnic groups at risk, such as Haitian Americans and other immigrant populations.

### 4.5. Communication Channels During an Extreme Heat or Other Weather Emergency

Many local and national governments offer alerts to their communities in advance of and during climate-change-related health emergencies, such as wildfires, extreme storms, or extreme heat events [27]. Our results confirm a willingness among patients in climate-vulnerable neighborhoods to receive alerts and indicate a preference for receiving text messages. However, other methods were popular as well, so emergency planners should consider offering alerts via multiple channels so as to reach every vulnerable individual. An intriguing question for future research is whether health alerts originating from a person’s personal clinician could be more effective than government-issued alerts.

### 4.6. Low Rates of Teaching and a Role for the Waiting Room

A goal of our study was to assess whether the medical office waiting room could be an effective site for teaching about heat illnesses and other topics. A few studies on this question conclude that clinicians rarely discuss climate change with patients due to limited knowledge, time constraints, uncertainty about what are effective teaching methods, and political sensitivities [13,14]. According to Boland et. al, 64% of primary care physicians believe climate change is affecting patient health, while only 17% are comfortable counseling patients on climate-change-related health topics [15]. Boland et al. found that only 33% of physicians in their study reported that they are well-informed or very well-informed on these topics. Furthermore, Boland et al. discuss how the time constraints of the patient visit limit a primary care physician’s ability to discuss climate-related health topics.

A 2021 report described the use of electronic health records of 21,010,780 U.S. primary care visits to determine that the average primary care appointment lasts only 18.0 min, with much of that time consumed by data entry rather than in conversation with patients [28].

Another impediment is the perception that the topic of climate change is politically controversial for some patients. Lewandowski et al. did not find that a brief discussion of climate change generated anger against the clinician, even for self-described Republican Party members, but it is still likely that many clinicians would be hesitant to risk disrupting the patient visit or risk losing the trust of their patients by broaching this topic in the examination room [14].

The waiting room is less burdensome for the office staff and clinicians as a location to teach about heat illness and other climate-related health topics. Our study was conducted using printed teaching materials which patients were to take home to read; we carefully excluded the clinical staff, nurses, and doctors from any involvement. We found that patients are highly appreciative of receiving information in the waiting room. Brochures and one-page fliers were both well-received. Video screen messages and posters are also teaching options that are liked. Interestingly, and similarly to the findings of Ragavan et al. [13], 34% of patients also desired a short discussion with their doctor or nurse, underscoring the need for clinicians to have some training on heat illness and similar topics.

We find that even simple, low-cost interventions, such as brochures, can lead to behavior changes when provided by trusted health professionals. For example, brochures can be printed in bulk for as little as USD 0.06 each (personal observation, T.L.S.).

### 4.7. Study Limitations

There are several important limitations to this study. The first is our small sample size of ninety-one drawn from one U.S. city. Our educational materials concerned only heat-related illness and not other topics of climate change and health, environmental health, or climate resilience. We believe that the results are reasonably representative of community opinions because the survey methodology was anonymous and confidential and because the respondents represent a wide range of ages, genders, and ethnicities.

Our small sample size and aspects of the protocol design do not permit detailed statistical comparisons among the variables that we studied, and this was a missed opportunity. We sought to survey climate-vulnerable communities, and this meant conducting our study in many small, free, and variably structured clinics where it was not possible to define the overall size of clinic populations. Therefore, we were unable to include the population’s statistical universe in our analyses.

We caution future investigators that we found obtaining completed surveys to be challenging. Our low response rate occurred despite significant steps taken, including buy-in from clinic staff; the use of trained volunteers to deliver the educational materials; bilingual printed teaching materials, instruction cards, and surveys; a gift bag given to each patient; and the offer of a USD 10 gift card. One of our authors (T.L.S.) found in a previous failed study that asking the office staff to distribute teaching materials to waiting room patients was not successful, and this is, in part, why we do not recommend asking busy office staff or clinicians to play a significant role in climate and health education.

As noted above, the behavioral changes reported by the respondents are non-quantitative, self-reported, and not verified over time.

## 5. Conclusions

Health professionals are highly trusted sources of health information. We conclude that patients who reside in heat-vulnerable neighborhoods are willing to receive in the medical office waiting room educational materials on heat-related illness and severe weather. Printed brochures were favored statistically by patients in this small study, but other teaching methods that are feasible for waiting rooms also rated well, including single-page printed fliers, posters, and video screens. Notably, several digital teaching options were far less attractive for our patient population. In addition, a third of patients expressed that they would like a short discussion from the doctor or nurse. We conclude from this short-term study that printed teaching materials may improve decisions in the home that impact human health. The medical office waiting room appears to be an accepted, time-efficient, and effective location for health professionals to communicate to patients and communities critically needed knowledge on climate change and health. Additionally, medical offices could play a critical role in supporting government agencies to communicate to their patients during climate-related health emergencies.

This work paves the way for further research into the effectiveness of the medical waiting room as a site to teach topics of environmental health and climate change resilience and, specifically, to study this question with other patient populations; to further compare the effectiveness of printed and digital resources across different ages, socioeconomic statuses, and ethnicities; and to look at whether this teaching has long term impacts that may improve health outcomes. Another important question to ask in future investigations is how offering teaching materials on climate change and health in waiting rooms is received by clinicians and other office staff members.

## Data Availability

The original contributions presented in this study are included in the article/Supplementary Materials. Further inquiries can be directed to the corresponding author.

## Supplementary Materials

The following supporting information can be downloaded at: https://www.mdpi.com/article/10.3390/ijerph22030434/s1, Supplementary Material S1: Qualtrics Survey: Heat Education Survey Florida 2023; Supplementary Material S2: Survey results raw data
