# Supplementary material for: Heat Illness and Extreme Weather Health Literacy: Communication Preferences and Effectiveness for Patients Living in Climate-Change-Vulnerable Communities
Source: Int J Environ Res Public Health. 2025 Mar 14;22(3):434. doi: 10.3390/ijerph22030434 (PMC11941823; doi:10.3390/ijerph22030434)
Supplement: Supplementary file 1 [file ijerph-22-00434-s001.zip › Sack et al 2025 Supplementary Materials S1. Qualtrics Survey Heat_Education_Survey_Florida_2023.pdf]

Sack et al 2025

Supplementary Materials

A. Qualtrics Survey used in this study “Heat Education Survey Florida 2023”

Start of Block: Default Question Block

Q1
You are invited to participate in this five-minute research survey about the printed information that you have been given on heat-related illnesses. This survey has been approved by the Institutional Review Board at Florida International University to help protect your privacy. **Purpose:** The purpose of this study is to improve how doctors and nurses inform patients about heat-related illnesses. **Procedures:** You will be asked to complete a short online survey. **Duration:** This will take about five minutes. **Risks and Discomforts:** There are no physical risks to participating. **Costs:** There are no costs to you for participating in this survey
Benefits: You have received free printed information about heat-related illnesses and will receive at **$10 Walmart gift card** after completing the survey.
Alternatives: There are no alternatives other than to not complete the survey.
Participation: Your participation in this study is voluntary. You have the right to withdraw your consent, to discontinue participation at any time, and to refuse to answer any questions.
Confidentiality: Your responses will be kept private. You may provide your email address in order to receive a $10 Walmart gift card but all email addresses will be deleted once the gift cards have been sent. Results of this survey may be presented at scientific or professional meetings, or published in scientific journals; your identity will never be included. **Researcher Contact Information:** If you have any questions, please email one of the Principal Investigators: Dr Cheryl Holder (clholder@fiu.edu) or Dr. Todd Sack (tsack@fiu.edu).
Institutional Review Board Contact Information: If you wish to talk with someone about your rights or about ethical issues with this study, you may contact the FIU Office of Research Integrity: 305-348-2494 or ori@fiu.edu.
Age to Participate: You must be at least 18 years old to take the survey.
Agreement to Participate: I have read the information in this consent form and agree to participate. **I am typing my age in the box below. By doing so, I am providing my informed consent:**

____

Skip To: End of Survey If Condition:

Q19 You were given a Survey Instruction Card. Please type below the Survey Number found at the top right of the card:

____

Page Break

Q4
Next, we'll ask four quick questions about you.
What is your gender?

- ○ Female (1)
- ○ Male (2)
- ○ Non-binary / Gender fluid / Other (3)
- ○ Prefer not to say (4)

Q5 How would you describe yourself? You may choose more than one:

- ☐ African American / Black (2)
- ☐ Asian / Pacific Islander (3)
- ☐ Caucasian / White (6)
- ☐ Haitian / Haitian American (9)
- ☐ Hispanic / Latin American (1)
- ☐ Native American / American Indian / Indigenous peoples (4)
- ☐ Middle Eastern / Arab American (5)
- ☐ Other (7)
- ☐ I prefer not to say (8)

Page Break

*

Q6 What is the zip code for where you live?

____

Q18 How old are you?

- ○ 18-29 years old (1)
- ○ 30-39 years old (2)
- ○ 40-49 years old (3)
- ○ 50 -59 years old (4)
- ○ 60 years old or older (5)

End of Block: Default Question Block

Start of Block: Block 1

Q7 Now, we will ask about the brochure that you were given, "Surviving Extreme Heat". Have you read part or all of this brochure?

- ○ Yes, I have read part or all of this brochure. (1)
- ○ No, I have not read any of this brochure. (2)

Skip To: End of Block If Now, we will ask about the brochure that you were given, "Surviving Extreme Heat". Have you read... = No, I have not read any of this brochure.

Q8 About the brochures, "Surviving Extreme Heat":

	I agree (1)	I somewhat agree (2)	I somewhat disagree (4)	I disagree (5)	I have no opinion (6)
I am glad that my medical office provides this information. (1)	o	o	o	o	o
I learned something new from this brochure. (2)	o	o	o	o	o
This brochure is easy to read. (3)	o	o	o	o	o
I will make new decisions in my home or for my family because of this brochure. (6)	o	o	o	o	o
This brochure is too long. (10)	o	o	o	o	o
I will share this brochure with a family member or friend. (11)	o	o	o	o	o

End of Block: Block 1

Start of Block: Block 3

Q11 You were give a two-sided printed handout. One side of has the title, "Heat Stress Overview". Have you read part or all of this?

○ Yes, I have read part or all of this side of the handout. (1)

○ No, I have not read any of this side of the handout. (2)

Skip To: End of Block If You were give a two-sided printed handout. One side of has the title, "Heat Stress Overview". Ha... = No, I have not read any of this side of the handout.

Q12 About the handout, "Heat Stress Overview":

	I agree (1)	I somewhat agree (3)	I somewhat disagree (5)	I disagree (4)	I have no opinion (6)
I am glad that my medical office provides this information. (1)	○	○	○	○	○
I learned something new from this handout. (2)	○	○	○	○	○
This handout is easy to read. (3)	○	○	○	○	○
I will make new decisions in my home or for my family because of this handout. (6)	○	○	○	○	○
This handout is too long. (7)	○	○	○	○	○
I will share this handout with a friend or family member. (8)	○	○	○	○	○

End of Block: Block 3

Start of Block: Block 4

Q13 You were give a two-sided printed handout. One side of has the title, "Staying Safe in Hot Weather". Have you read part or all of this?

- ○ Yes, I have read part or all of this side of the handout. (1)
- ○ No, I have not read any of this side of the handout. (2)

Skip To: End of Block If You were give a two-sided printed handout. One side of has the title, "Staying Safe in Hot Weathe... = No, I have not read any of this side of the handout.

Q14 About the handout, "Staying Safe in Hot Weather":

	I agree (1)	I somewhat agree (2)	I somewhat disagree (4)	I disagree (5)	I have no opinion (6)
I am glad that my medical office provides this information. (1)	○	○	○	○	○
I learned something new from this handout. (2)	○	○	○	○	○
This handout is easy to read. (3)	○	○	○	○	○
I will make new decisions in my home or for my family because of this handout. (6)	○	○	○	○	○
This handout is too long. (7)	○	○	○	○	○
I will share this handout with a friend or family member. (8)	○	○	○	○	○

End of Block: Block 4

Start of Block: Block 2

Q17 Please tell us of any new decisions you may make in your home or for your family because of the brochure or handout you were given. **Is there anything else you would like to tell us?**

____

____

____

____

____

End of Block: Block 2

Start of Block: Block 5

Q15 Please tell us how you would like your health professionals to tell you about extreme heat and its health risks. **Select as many as you wish.** Choose "None" if you do not want to learn about this from the medical office or have no opinion.

- ☐ Waiting room brochures to take home (1)
- ☐ Waiting room posters (2)
- ☐ Waiting room videos (3)
- ☐ Waiting room 1-page handouts to take home (7)
- ☐ Brochures I can read on my computer or phone at home (8)
- ☐ A short discussion by the doctor or nurse (4)
- ☐ Information printed on my office visit summary (5)
- ☐ A newsletter emailed to me from the medical office (6)
- ☐ With a phone App (11)
- ☐ On social media such as Instagram, Facebook, Twitter, or YouTube (12)
- ☐ Other: (10) ____
- ☐ None or I have no opinion about this. (15)

Q16 In a health emergency such a severe storm, extreme heat event, or wildfire, would you like your health professional or local health department to alert you? **Choose all that apply**:

- ☐ No, I would not want to be contacted. (1)
- ☐ Yes, I would want to be alerted with a phone call. (2)
- ☐ Yes, I would want to be alerted with a text message. (3)
- ☐ Yes, I would want to receive an email. (4)
- ☐ Yes, with social media such as Instagram, Facebook, Twitter. (16)
- ☐ Yes, with a phone App created to alert people during a health emergency. (17)
- ☐ I do not know or have no opinion. (5)

Page Break

End of Block: Block 5
